# First Case of Topical 5-FU Therapy for Cutaneous Metastasis in a Patient with Colon Cancer

**DOI:** 10.14744/ejmo.2020.81980

**Published:** 2020-03-12

**Authors:** Gulrayz Ahmed, Sylvia Alarcon Velasco, Layla Van Doren, Muhammad Wasif Saif

**Affiliations:** 1 Department of Hematology and Oncology, Tufts Medical Center, Massachusetts, USA; 2 Northwell Health Cancer Institute, Donald and Barbara Zucker School of Medicine at Hofstra, Feinstein Institute for Medical Research, Lake Success, NY, USA

**Keywords:** Adenocarcinoma, cutaneous metastasis, colon cancer, fluorouracil imiquimod, skin-directed treatment, synergy, synergy of topical and systemic treatments, topical treatment, 5-FU

## Abstract

Cutaneous metastasis from colon cancer is rare, occurs in less than 6% of patients and its associated with poor prognosis. Most often it presents in the abdomen, inguinal or perineal regions, supraclavicular area, and less commonly on the face, neck, scalp, and prior surgical sites. We present a case of a 41-year-old female with colon cancer who developed cutaneous metastases to the scalp, and was treated with topical 5-FU and radiation therapy. Treatment options for cutaneous metastases usually include systemic therapy, topical chemotherapy, surgical excision, or radiation. Our case is probably the first report who was treated with topical 5-FU in addition to radiation therapy. This treatment modality is easy to use and we would recommend clinical trials to be conducted to further study the use of topical 5-FU.

Cutaneous metastasis from colon cancer is rare, occurs in less than 6% of patients and its associated with poor prognosis.^[1]^ Most often it presents in the abdomen, inguinal or perineal regions, supraclavicular area, and less commonly on the face, neck, scalp, and prior surgical sites.^[2]^ Treatment options include systemic therapy, topical chemotherapy, surgical excision, or radiation. We present a case of a 41-year-old female with colon cancer who developed cutaneous metastases to the scalp, and was treated with topical 5-FU and radiation therapy.

## Case Report

The patient is a 42-year-old Caucasian woman who presented with significant weight loss, progressive right-side abdominal pain and distention for close to a year. Family history of a maternal aunt with ovarian cancer diagnosed at the age of 44 as well as another maternal aunt with breast cancer. Initial imaging, CT scan revealed a 30 cm mass within the distal small bowel, an 11 cm complex cystic mass posterior to the uterus, a 16 ×7 cm liver mass and numerous small nodules throughout the liver and lungs. Her liver biopsy revealed adenocarcinoma of colonic origin. Immunohistochemistry was positive for pankeratin, CK20, and CDX-2, and negative for TTF-1, ER, PR, CK7 and CA-125. Tumor markers were elevated with CEA of 409.7 ng/mL (normal range 0.0–4.9 ng/mL), CA-125 of 70.6 U/mL (normal range 0–34 U/mL) and CA 19–9 of 1726 U/mL (normal range <34 U/mL). She underwent diagnostic laparoscopic transverse colostomy placement to relieve bowel obstruction. Intraoperative peritoneal fluid pathology was negative for malignant cells. Further imaging with MRI abdomen showed a complex cystic right adnexal lesion most consistent with primary ovarian cancer. She was evaluated by gynecologic oncology who recommended against biopsy due to high risk of rupture because of its cystic nature. Genetic analysis of the colon cancer showed KRAS and TP53 mutations. No loss of nuclear expression of mismatch repair proteins. She had homozygous wild-type polymorphism for UGT1A1 (UDP glucuronosyltransferase family 1 member A1).

In the setting of stage IV colorectal cancer and suspicion of ovarian cancer, she was started on chemotherapy with FOLFIRINOX (5-FU without bolus, leucovorin, irinotecan and oxaliplatin) and completed 12 cycles. Following that she underwent total abdominal hysterectomy and bilateral salpingo-oophorectomy for suspected ovarian cancer. The pathology was consistent with metastatic adenocarcinoma of colonic origin. Her follow up CT scan showed progressive metastatic disease. She initiated second line treatment with FOLFIRI (Irinotecan with 5-FU and folinic acid) with bevacizumab.

**Figure 1.**
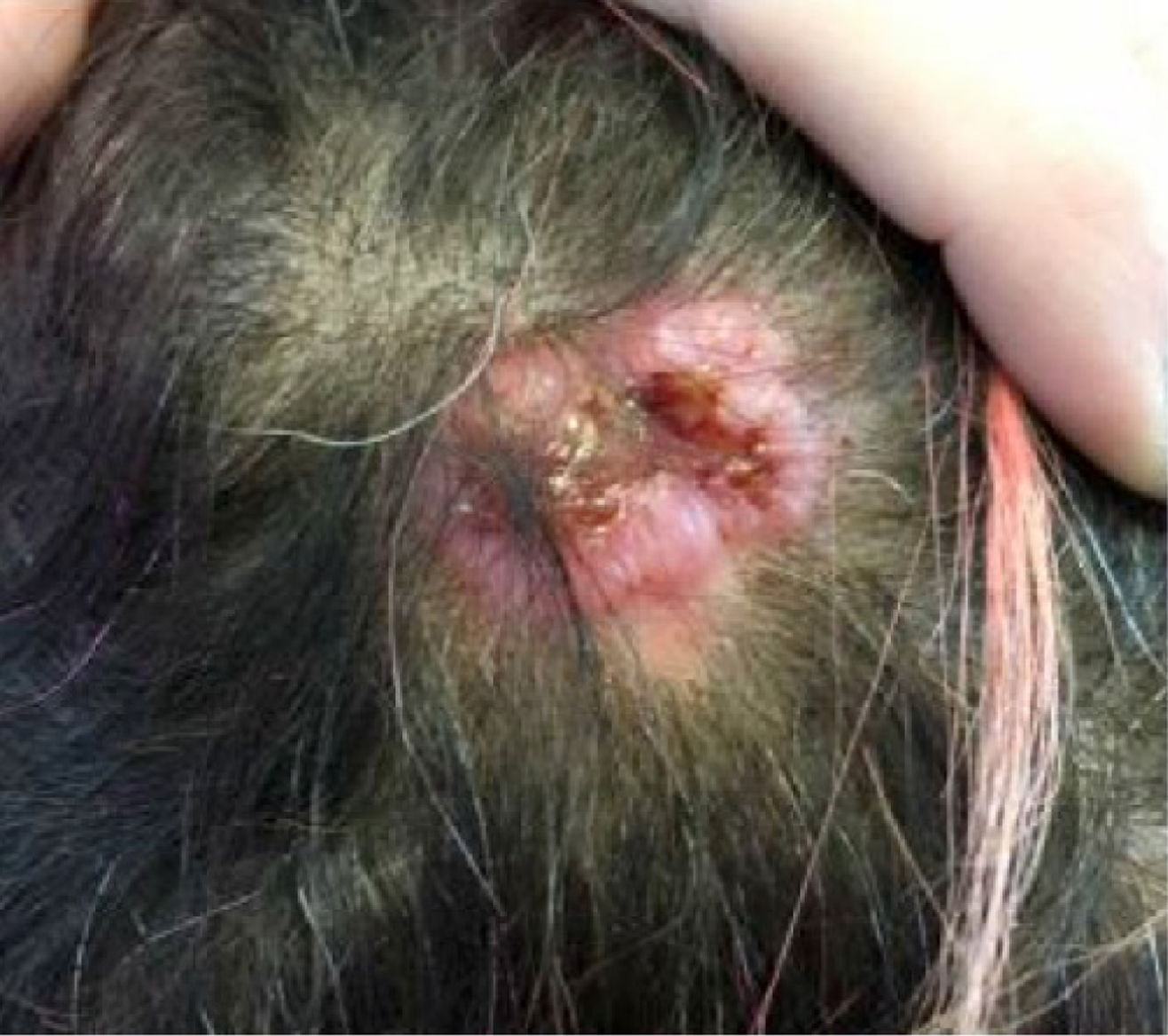
Initial presentation of the scalp lesion.

**Figure 2.**
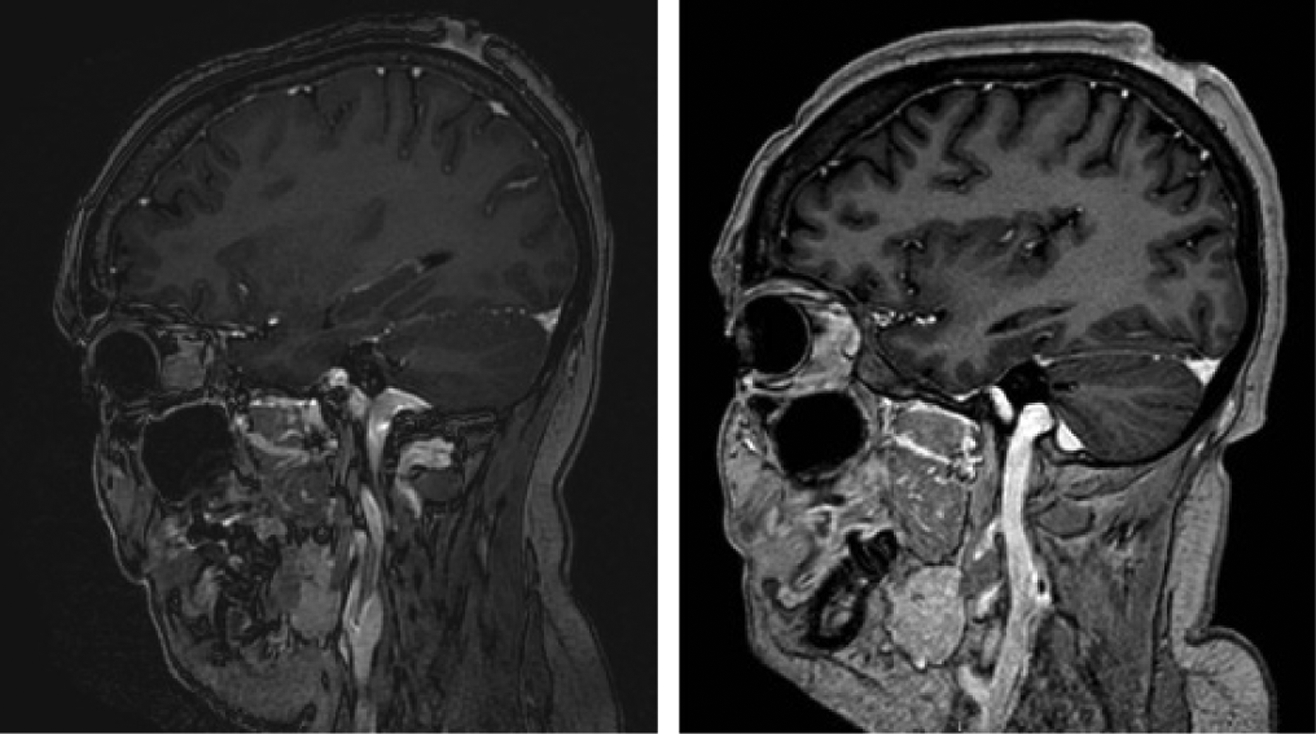
MRI scan of headshowing the location and depth of scalp mets.

During this treatment, she noticed a non-healing cyst-like lesion on the posterior right parietal scalp, which was slowly growing over one year (Fig. 1). The lesion was sometimes painful with spontaneous serosanguineous discharge. She was evaluated by dermatology followed by resection of her right parietal scalp 2.5×2.2 cm lesion. An MRI of the head (Fig. 2) was done to assess the depth of the lesion and deep tissue involvement. The pathology was consistent with adenocarcinoma of colonic origin with positive positive. Palliative radiation therapy (PRT) was recommended in case of worsening symptoms such as bleeding or pain. Patient received 24 Gy in 3 fractions.

**Figure 3.**
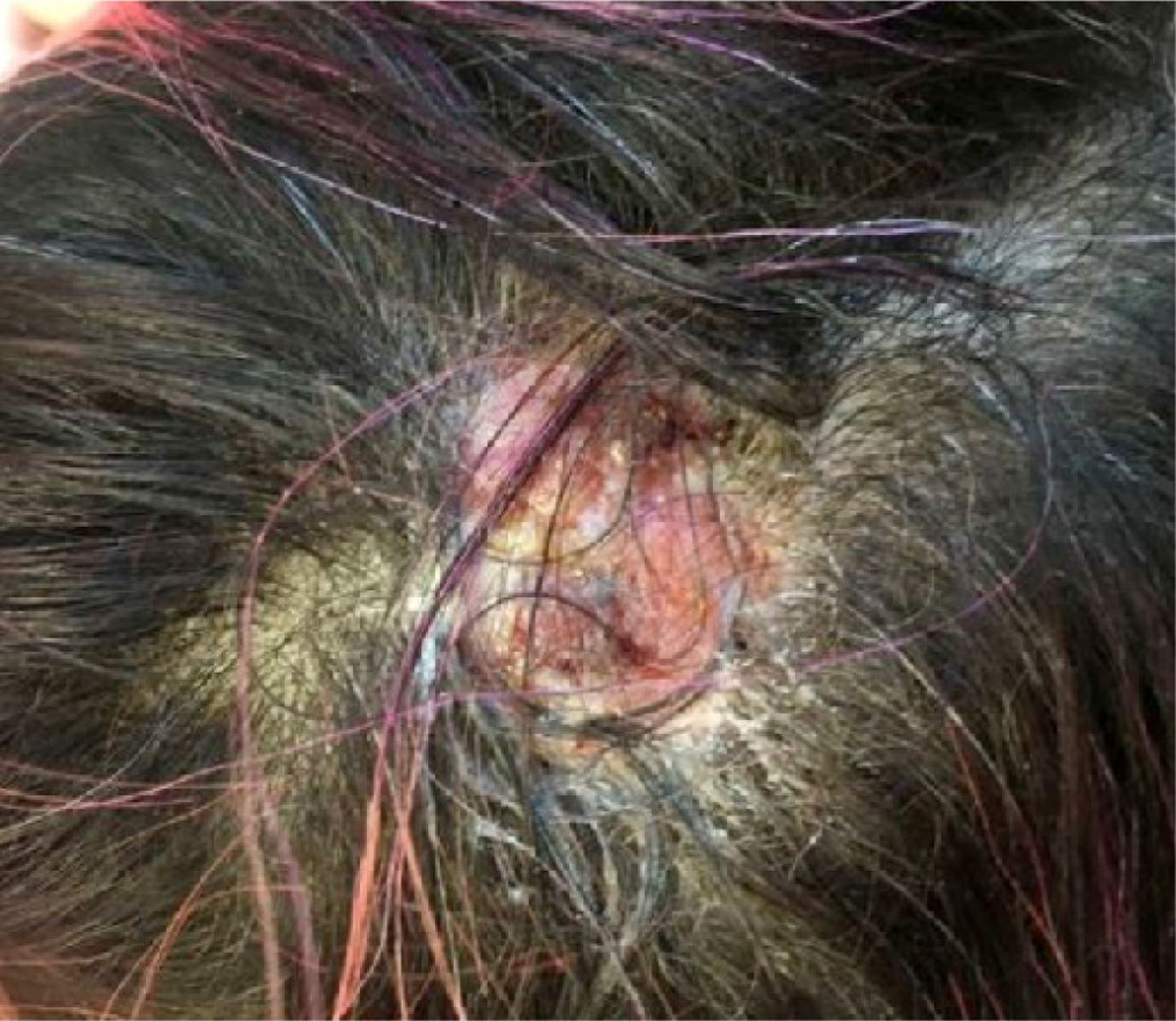
MRI scan of headshowing the location and depth of scalp mets.

Due to progression of the scalp lesion, topical 5-FU twice daily was started. Her scalp lesion started showing signs of healing with scab formation starting at week 4 with decreased in size and dramatically improved in 8 weeks (Fig. 3).

Five months after initiation of topical 5-FU the patient reported increasing frequency of local symptoms including a sharp stabbing pain, increased headache and tender to touch with deep aching. She also developed a palpable small nodule in the right sub-occipital region with concern for a new metastatic lesion. MRI head revealed a soft tissue lesion in the right parietal scalp, measuring 2.4 cm (previously 1.6 cm) with a new 1 cm enhancing soft tissue lesion in the right sub-occipital scalp.

Despite the numerous systemic treatments including FOLFIRI with bevacizumab, Regorafenib, MIRI (mitomycin-C, irinotecan), FOLFOXIRI (folinic acid, 5-fluorouracil, oxaliplatin and irinotecan) and bevacizumab and topical 5-FU her scalp lesion progressed. Palliative radiation therapy was started. Following radiation therapy with concurrent 5-FU her scalp lesions remained stable and her symptoms improved.

Her overall systemic disease continued to progress while on therapy and after significant decline in functional status, the patient was transitioned to comfort measures.

## Discussion

Colon cancer rarely metastasizes to the skin and occurs in less than 6% of patients.^[1,2]^ Cutaneous metastases occur concurrently with widespread metastatic disease to other sites such as liver, peritoneum and lung.^[3,4]^ Most frequent sites of cutaneous metastasis are the abdomen, inguinal or perineal regions, and prior surgical sites, and occur with less frequency on the face, neck and scalp.^[5,6]^ Adenocarcinoma has the highest rate of cutaneous metastasis compared to other histologic subtypes. The skin metastasis can appear as sessile, pedunculated, single or multiple nodules, as a mass with ulceration, or as a cyst.

The mechanism is thought to be secondary to lymphatic or hematogenous spread, local extension of the tumor, and surgical implantation during resection of the primary lesion.

Cutaneous metastases have been associated with a poor prognosis, with overall survival approximately 12–18 months.^[2–10]^ Aravind et al.^[10]^ in their article have divided cutaneous metastasis cases in to two groups:

**Group 1:** One who primarily present with cutaneous manifestations with primary not identified
**Group 2:**This group is the one which has been treated with resection of the primary tumor and is being followed up by medical oncology.

The investigators pointed out that the former group is usually associated with more poor outcomes and other visceral involvement.

No clear guidelines exist for treatment of cutaneous metastasis with primary go to treatment being wide excision of the lesion. Systemic chemotherapies and local radiation have also been used but no topical treatment options have been ever reported. Radiation is mostly used in palliation for painful or bleeding lesions but with modest results.[3–9] Table 1 summarizes all case reports published on PubMed with English abstracts since 2013 and treatment options utilized. Overall, the goal of treatment is symptomatic relief. No topical chemotherapy has been previously reported. Our patient was prescribed topical 5-FU (5%) once daily and initially her lesion responded as shown above in the picture, but eventually her overall disease progressed though being on systemic chemotherapy and eventually passed away after a total treatment time of 18 months.

We searched the medical literature with diligence and finally found two more cases who received topical 5-FU for skin metastases in patients with breast cancer.^[22]^ Among a case series, two patients used topical 5-FU. When used alone, 5-FU reduced bleeding and drainage of lesions and when combined with cryotherapy and systemic therapy, rapidly decreased tumor burden. The authors concluded that combined treatment with cryotherapy and topical 5FU is superior to cryotherapy alone, suggesting that 5-FU induces an antitumor activity independent of cryotherapy. Similar to imiquimod, the authors also suggested that the dramatic response in both patients is in part owing to a favorable immune milieu induced by 5-FU that synergizes with systemic therapies.

Our case showed that topical 5-FU can result in partial regression or local control when used as monotherapy or in combination with other treatment modalities like in our patient along with radiation therapy. Side effects include irritation to the applied area and burning sensation. This treatment modality is feasible with a favorable side effect profile; however, the role of topical 5-FU needs to be further investigated, including tests to characterize the antitumor responses elicited by 5-FU.

## Conclusions

Cutaneous metastasis is of rare occurrence. In literature they occur either as primary presentation or develop while he patient has been treated and is being followed up appropriately. No specific guidelines exist for treatment options of cutaneous metastasis with wide surgical excision being the most reported treatment. Topical 5-FU can be used as a potential treatment but clinical trials need to be conducted.

## Figures and Tables

**Table 1.** Summary of previously published Case Reports with cutaneous metastases associated with CRC

Author et. al	Age	Gender	Site of cancer treatment	Phase of Mets	Site of skin systemic Mets	Presence of	Treatment	Outcome (survival after cut Mets diagnosed)
Ha JY et. al; 2016^[6]^	78	Male	Ascending Colon	Follow up	Right Parietal Scalp	Lung	Systemic Capecitabine	Poor
Góes HF et. al; 2016^[11]^	76	Female	Descending Colon	Initial	Right Parietal Scalp	None	Surgical Excision and FOLFOX	Good
Udkoff J et. al; 2016^[12]^	56	Male	Colon	Follow up	Scrotum	None	FOLFIRI	Fair
Dehal A et. al; 2016^[13]^	47	Male	Rectal	Follow up	Genital area and perineum	Local lymph nodes and vessels	Local Radiation	Good
Reusser NM et. al; 2015^[14]^	58	Male	Rectal	Follow up	Right Flank	NA	Palliative	Poor
Abt NB et. al; 2015^[15]^	67	Male	Sigmoid Colon	Initial	Pelvis and Scrotum	Local Lymph nodes and skin	Patient declined treatment	Poor
Sheets N et. al; 2014^[16]^	78	Male	Ileocecal Valve	Initial	Left Scapula	None	Surgical Excision	Good
de Miguel Valencia MJ et. al; 2013^[17]^	55	Male	Rectal	Follow up	Multiple subcutaneous lesions on face, axilla, chest, flank and lower extremities	Lung and Liver	None	Poor
Nesseris 1 et. al; 2013^[4]^	80	Male	Ascending Colon	Follow up	Abdominal Surgical Scar	None	Surgical Excision	Good
Hashimi Y et. al; 2013^[3]^	70	Male	Rectal	Follow up	Right Cheek	Lung	Surgical Excision	Fair
Aravind Bet.al; 2013^[10]^	61	Female	Rectal	Follow up	Scalp (recurrent)	Lung	Surgical resection	Good
Balta AZ et.al; 2013^[18]^	84	Female	Rectal	Initial	Left Occiput	None	Chemotherapy and Radiation	Poor
Relies Det.al; 2012^[19]^	55	Male	Sigmoid	Follow up	Right upper lip	Liver	Excision	Poor
Balta 1 et.al; 2012^[20]^	46	Male	Rectal	Follow up	Anogenital region	Local	Not resectable. Patient denied chemo	Unknown
Nguyen VX et. al; 2012^[21]^	65	Male	Caecum	Initial	Right Flank	None	Systemic Capecitabine and cyclophosphamide, progressed then on FOLFOX	Poor
